# Causes and Consequences of COVID-19-Associated Bacterial Infections

**DOI:** 10.3389/fmicb.2021.682571

**Published:** 2021-07-20

**Authors:** Jennifer M. Farrell, Conan Y. Zhao, Keiko M. Tarquinio, Sam P. Brown

**Affiliations:** ^1^School of Biological Sciences, Georgia Institute of Technology, Atlanta, GA, United States; ^2^Center for Microbial Dynamics and Infection, Georgia Institute of Technology, Atlanta, GA, United States; ^3^Interdisciplinary Graduate Program in Quantitative Biosciences, Georgia Institute of Technology, Atlanta, GA, United States; ^4^Pediatric Critical Care Medicine, Department of Pediatrics, Emory University School of Medicine, Children’s Healthcare of Atlanta, Atlanta, GA, United States

**Keywords:** COVID-19, bacteria, pneumonia, secondary infection, co-infection, ICU

## Abstract

The COVID-19 literature highlights that bacterial infections are more common in fatal cases than recovered cases. If bacterial infections drive mortality in COVID-19, this has clear implications for patient management. However, it is possible that the enrichment of bacterial infections in COVID-19 fatalities is simply a by-product of late-stage pathology, leading to different advice for patient management. To address this question, we review current knowledge on bacterial infections in COVID-19, assess information from past viral respiratory pandemics, and simulate alternate causal models of interactions between virus, bacteria, and mortality in COVID-19. From these models, we conclude that currently available data are not sufficient to discriminate between these alternate causal pathways, and we highlight what data are required to determine the relative contribution of bacterial infection to COVID-19 morbidity and mortality. We further summarize the potential long-term consequences of SARS-CoV-2 infection.

## Introduction

### Bacterial Secondary Infections in COVID-19: What We Know

Of the available studies that address bacterial infection in COVID-19, few specify critical information including diagnostic criteria for bacterial infection, timing of sample collection, or even the identity of bacterial species, making comparison across studies difficult. Of the available data, however, bacterial infection (typically diagnosed as secondary pneumonia or bacteremia) at some point during COVID-19 hospitalization has been reported in about 6–15% of patients where bacterial infection was assessed (Rawson et al., 2020). In a meta-analysis of bacterial infection in people hospitalized with COVID-19, Langford et al. (2020) found that an average of 8% of patients in all included studies were found to have coinfections or secondary bacterial infections. However, it is difficult to distinguish between viral and bacterial pneumonia, even during normal hospital circumstances; in a study of five US hospitals between 2010 and 2012, no specific microbiological etiology was determined for 62% of patients with pneumonia (Jain et al., 2015). Furthermore, collection of respiratory samples from COVID-19 patients is complicated by the high-risk nature of aerosol-generating procedures^1^. It is therefore possible that bacterial respiratory infections are going under-detected in patients hospitalized with COVID-19. Few papers report the species identity or time of specimen collection, making it impossible to determine whether any patients presented with bacterial infection at the time of hospital admission. However, in papers that do report recovered organisms, gram-negative opportunists such as *Acinetobacter baumannii*, *Klebsiella pneumoniae*, and *Pseudomonas aeruginosa* are reported (Chen et al., 2020; Yang et al., 2020). These species are frequently seen in nosocomial infections, especially ventilator-associated infections (Chi et al., 2012).

### Are Bacterial Secondary Infections Important Causal Drivers of Mortality?

**FIGURE 1:**
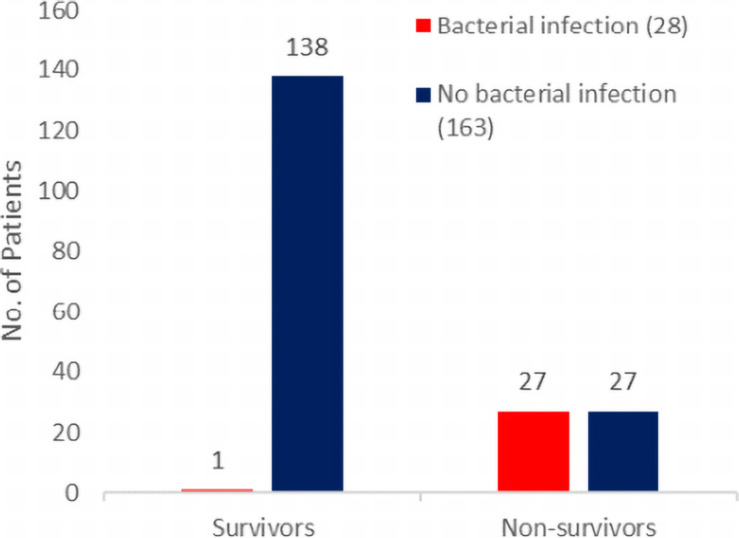
Bacterial secondary infections are more common in fatal COVID-19 cases. Patient outcomes (survival to hospital discharge versus death during hospitalization) and bacterial infection status (red for infected and blue for non-infected) from the 191-patient cohort of Zhou et al. (2020) consist of all adult inpatients with laboratory-confirmed COVID-19 from the Jinyintan Hospital and Wuhan Pulmonary Hospital (Wuhan, China) who had been discharged or had died by January 31, 2020.

One line of evidence that indicates the importance of bacterial infections in COVID-19 are studies that stratify on patient outcomes. Of articles that list bacterial infection rates among COVID-19 patients, only a few give details on the outcomes of those patients. In a 191-patient retrospective cohort study in Wuhan, China, Zhou et al. found that bacterial infections (bacteremia and pneumonia) were more common in fatal COVID-19 cases, compared with recovered cases (Figure 1): 28/191 (15%) of patients had a culture-positive bacterial infection, and of these patients, all but one died (Zhou et al., 2020). Half of non-survivors (27/54) experienced a bacterial infection compared with only 1% of survivors (1/137) (Zhou et al., 2020). Similar patterns of higher incidence of bacterial infections among patients who died have been reported in later studies in Wuhan (Wang et al., 2020) and in Spain (Ferrando et al., 2020). Beyond COVID-19, similar patterns have been reported for influenza – notably a retrospective analysis of the 1918 pandemic where the ubiquity of bacterial involvement in fatal cases (seen from preserved histology samples) gave support to the conclusion that upward of 95% of the mortality was directly attributable to secondary bacterial pneumonia (Morens et al., 2008).

**FIGURE 2:**
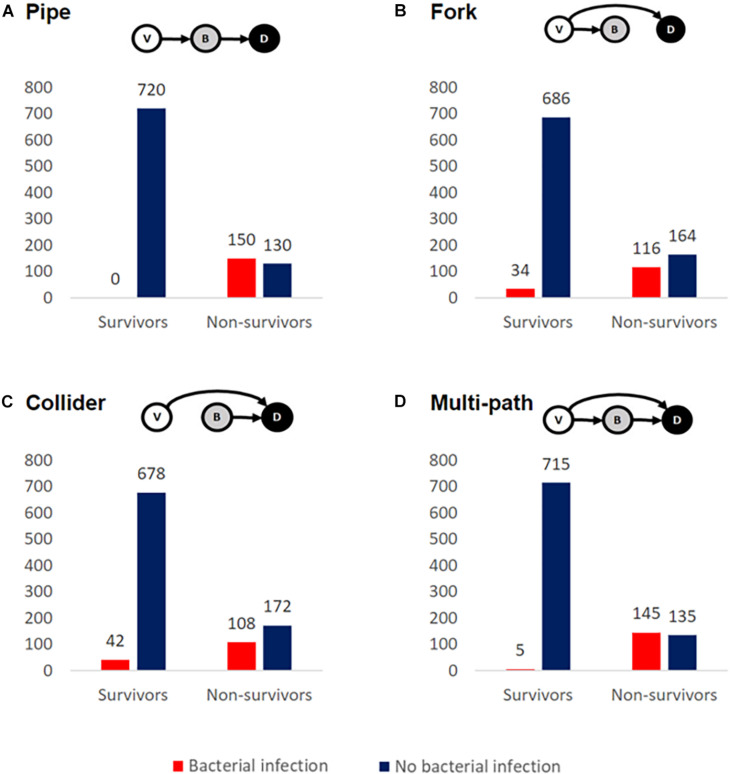
Alternate causal models are consistent with enrichment of bacterial secondary infections in fatal COVID-19 cases. We simulated cohorts of 1,000 hospitalized COVID-19-positive cases, constrained by the data of Zhou et al. (2020) ensure that 28% overall mortality and 15% of cases have culture-positive bacterial infection. The inset schematics summarize four distinct causal models for relationships between viral severity, *V*, bacterial severity, *B*, and patient death, *D*. In all scenarios, the mortality rate is greater when conditioned on bacterial infection, regardless of whether *B* is the sole direct cause of death **(A)**, a partial cause of death **(C,D)**, or not a direct cause of death **(B)**.

The higher incidence of bacterial infections in fatal cases is consistent with bacterial involvement in mortality but does not rule out various alternative causal hypotheses, including a case where bacteria have no causal impact on mortality (given bacterial growth and mortality share a common viral cause). Elucidating the causal relationships between SARS-CoV-2 infection, bacterial coinfection/secondary infection, and mortality is critical for the rational identification of effective treatment plans for patients with COVID-19. To illustrate this inferential challenge, we build a simple simulation model to examine alternate minimal causal paths of the within-patient interactions between virus, bacteria, and mortality (Figure 2). Our baseline assumption is that infected individuals vary in the degree of severity of viral infection, *V.* Empirically, variation in viral severity could encompass variation in viral load, and/or the extent of immunological perturbation caused by COVID-19. From this causal baseline, we build four canonical causal structures to relate variation in *V* to the likelihood of bacterial infection, *B*, and to the likelihood of death, *D* (Figure 2). We stochastically simulate each model structure for a cohort of 1,000 hospitalized patients under the constraint of the Zhou et al. article data proportions of bacterial infections (15%) and mortalities (28%) (Figure 1), and we ask how the distinct causal structures influence the statistical association between bacterial infection and mortality.

## Methods

We define random variables *B*, *V*, and *D* as measures of bacterial infection, viral severity, and patient mortality (Figure 2). For *B* or *D*, we further assume there exist threshold values above which a patient either has a clinically detectable bacterial infection or dies, respectively. For each modeled causal structure, we sample *B*, *V*, and *D* values for a population of 1,000 patients. For each causal structure, we additionally identify thresholds for *B* and *D* such that our observed infection and mortality likelihoods match the aggregate data from Zhou et al. (2020) (15% infected and 28% mortality). The model definitions are as follows.

### Pipe (Figure 2A)

*V* ∼ norm(0,1)
*B* ∼ norm(0,1) + *V*
*D* ∼ *B*

In the pipe, the causal impact of *V* on *D* is mediated by *B*. Here, *V* is drawn independently. *B* is dependent on *V*, and *D* is dependent on *B*.

### Fork (Figure 2B)

*V* ∼ norm(0,1)
*B* ∼ norm(0,1) + *V*
*D* ∼ *V*

In the fork, *V* is the common cause of *B* and *D*, but mortality is only a direct outcome of *V*. *V* is drawn independently. *B* is dependent on *V*.

### Collider (Figure 2C)

*V* ∼ norm(0,1)
*B* ∼ norm(0,1)
*D* ∼ *V* + *B*

In the collider, *V* and *B* are independent causes of *D*. Both *B* and *V* are drawn independently.

### Multi-Path (Figure 2D)

*V* ∼ norm(0,1)
*B* ∼ norm(0,1) + *V*
*D* ∼ *V* + *B*

In the multi-path model, *V* causes mortality both directly and indirectly (via *B*). *V* is drawn independently. *B* is dependent on *V*. *D* is dependent on both *V* and *B*.

In the Supplementary Material, we introduce tuning parameters *a*, *b*, and *c* to modulate relationships between *V*, *B*, and *D.* We vary these parameters and examine model consistency with the data of Zhou et al. (2020) (Supplementary Figure 1).

## Results

### Causal Model 1: The Pipe

In our first model (Figure 2A), we assume that the causal impact of *V* (viral severity) on *D* (patient death) is mediated by *B* (bacterial infection). In this model of cascading causes, viral severity promotes the establishment and growth of a bacterial infection, and this bacterial infection is then the primary cause of death. In this model, the virus alone does not directly cause mortality. It is important to underline that this and the following models are not mechanistically explicit, and multiple mechanisms are potentially consistent with each causal pathway. For instance, the pipe is consistent with virally induced processes that lead to increased *odds* of becoming bacterially infected [e.g., loss of lung barrier functions (Matheson and Lehner, 2020)] and/or increased *severity* of an established bacterial infection [e.g., via exhaustion or misdirection of immune defenses (Matheson and Lehner, 2020)]. Simulating this model structure, we find that bacterial infections are enriched among fatal cases (Figure 2A), as in the clinical data for hospitalized COVID-19 patients (Figure 1).

### Causal Model 2: The Fork

In our second model (Figure 2B), we again assume that viral severity drives bacterial infection, but we assign all mortality to be a direct outcome of *V*, with no effect of *B*. In other words, *V* is the common cause of *B* and *D*. As in the pipe model, the virus promotes bacterial infection through some mechanism [e.g., weakened immune system and/or increased exposure to pathogens in the intensive care unit (ICU) (Tosi et al., 2018)], but it is the virus alone that drives mortality; bacterial infection is simply a signpost of virally mediated death. In our simulation (Figure 2B), we again see the same qualitative pattern of bacterial infections enriched among fatal cases – despite the fact that *B* is not a direct cause of death.

### Causal Model 3: The Collider

Next, we model the case where *V* and *B* are independent causes of death (Figure 2C). The virus alone has some chance of causing death, and a bacterial infection has some chance of causing death, and these effects are additive. We again see an enrichment of bacterial infections among fatal cases.

### Causal Model 4: The Multi-Path

Finally, we consider the case where *V* causes mortality directly (*V* → *D*) and also indirectly via *B* (*V* → *B* → *D*) (Figure 2D). The virus may kill without bacterial infection and also potentiate a bacterial infection that then kills. In this model, we yet again see more bacterial infection in non-survivors. The multi-path causal model introduces the potential for multiple causal pathways connecting viral infection to death (direct and indirect) and so raises the more quantitative question of their relative importance.

To summarize, our simple epidemiological causal analysis demonstrates that we cannot simply infer from the suggestive pattern of data in Figure 1 that bacterial infections play a causal role in COVID-19 deaths – as alternate causal pathways can account for the same pattern in the data (Figure 2). Under all four causal structures, individuals with bacterial infection had a higher mortality rate than those without. In the pipe causal structure (Figure 2A), bacterial infection is the sole direct cause of death, and so consequently in our simulation of this model all individuals who died had a bacterial infection. We can therefore rule this structure out, as COVID-19 patients frequently die of viral complications without reported secondary bacterial infection. In contrast, the remaining three causal structures (Figures 2B–D) all generate patterns of data that reflect real-world observations (Ferrando et al., 2020; Wang et al., 2020; Zhou et al., 2020), while capturing very different biological assumptions on the relationships between viral infection, secondary bacterial infections, and death.

## Discussion

### Data and Approaches From Other Respiratory Viruses

Our survey of COVID-19 data illustrates that mortality data on bacterial secondary infections are scarce, and the available data do not allow discrimination of alternate causal models (Figure 2). What can we learn from other viral respiratory infections? Few papers address secondary bacterial infections in previous pandemic coronaviruses, with the existing papers listing mostly ESKAPE pathogens and atypical pathogens (*Mycoplasma pneumoniae*, *Chlamydia pneumoniae*, and *Legionella* spp.) at admission (Rawson et al., 2020).

Influenza secondary infections, on the other hand, are well-characterized. Historical cases and autopsy reports have led modern scientists to believe that upward of 95% of mortality from the 1918 flu pandemic was directly attributable to secondary bacterial pneumonia (Morens et al., 2008). Studies contemporaneous with the 1957–1958 flu pandemic estimated that ∼70–80% of fatal cases had bacterial pneumonia (Morens et al., 2008; Metersky et al., 2012), and studies from the 2009 H1N1 flu pandemic showed evidence of bacterial involvement in between 29 and 55% of hospitalized mortalities (Chieng Yeo et al., 2012). The original evidence for the importance of secondary bacterial pneumonia in influenza-related mortality came from the frequent isolation of bacteria from post-mortem lungs. Using this logic alone, we run into the same issues as we do in determining the importance of bacterial infection in COVID-19 outcomes: are bacterial secondary infections causing increased mortality (Figures 2A,C,D) or simply signposting it (Figure 2B)? However, in influenza, this evidence is bolstered by two critical lines of additional evidence: temporal clinical data and studies in laboratory models. These additional approaches lend support to a bacterial causal role. For example, a temporal analysis of clinical data showed that children hospitalized with severe bacterial (pneumococcal) pneumonia were more likely to have had influenza-like illness immediately prior to hospitalization (O’Brien et al., 2000), indicating a viral infection predisposing to later bacterial infection. Laboratory studies in both cell culture and mice show that the influenza virus can potentiate bacterial infection and pathogenicity (McCullers and Bartmess, 2003; Nickol et al., 2019). Such additional evidence could help clarify the question of attributable harm from COVID-19-associated bacterial infections by further discriminating among causal models in Figure 2. Temporal data tracking the timing of patient health decline and onset of bacterial infection could help clarify causality. If patients generally start to deteriorate directly prior to the onset of bacterial infection, it would support the theory that bacterial infection is a signpost of overall decline. If patients generally start getting worse directly *after* the onset of bacterial infection, it would support the idea that bacterial infection *causes* increased morbidity and mortality. Furthermore, experimental laboratory models of bacterial coinfection or secondary infection with SARS-CoV-2 will allow controlled investigation of causal paths and molecular mechanisms linking viral and bacterial dynamics with host outcomes.

### Long-Term Bacterial Consequences of COVID-19

#### Lung Damage and Future Infections

COVID-19 is a new disease, and we are only beginning to unravel the long-term complications of infection. It is possible that the damage done to the lungs and other organs in COVID-19 survivors may cause lasting damage and complications. Looking at other pandemic coronaviruses, one study found more than one-third of SARS patients had residual lung damage after 15 years (Zhang et al., 2020). Another study on MERS found that 36% of patients had lung damage on follow-up (between 32 and 230 days post-recovery) (Das et al., 2017). If lung damage does become a long-term complication of COVID-19, this may come with an increased bacterial infection risk long after patients have recovered from their initial COVID-19 infection.

#### Antibiotic Resistance

While the causal importance of secondary bacterial infections remains uncertain, precautionary medical practice currently involves frequent administration of antibiotics to COVID-19 patients (Rawson et al., 2020). Therefore, one possible longer-term outcome of the SARS-2-CoV pandemic is an increase in antibiotic resistance. In a secondary analysis of nine studies that included information on bacterial infections and antibiotic prescribing in COVID-19, Rawson et al. (2020) found that 72% (1450/2010) of hospitalized COVID-19 patients received empirical antibiotics, likely to rule out community-acquired bacterial infections and to prevent bacterial secondary infections. It is worth noting that the majority of the analyzed papers were from China early in the pandemic, and antibiotic prescribing will likely differ with time and location. As experience with COVID-19 increases, there is some evidence that antibiotic stewardship is becoming more of a priority. For example, the most recent guidelines from the UK National Institute for Health and Care Excellence indicate that antibiotics should only be used in COVID-19 cases if there is clinical suspicion of bacterial infection beyond symptoms of COVID-19 pneumonia (e.g., localized chest findings, a neutrophil count outside the normal range, lobar consolidation on chest imaging, and positive microbiology) (No authors listed, 2020).

## Conclusion and Future Research Questions

By assessing the possible roles of bacterial infection in COVID-19 mortality in light of current data, we find more questions than answers. Here, we outline what we see as critical questions that must be addressed to move forward in our assessment and management of potential bacterial infection risks in COVID-19:

1. What are temporal dynamics of bacterial infection and severity of symptoms during clinical COVID-19?
2. What are the impacts of COVID-19 on bacterial secondary infections and mortality in experimental animal models?
3. Do hospitalized COVID-19 patients with secondary bacterial infection die at a higher rate than non-COVID-19 patients who acquire the same bacterial infection?
4. What proportion of COVID-19-associated bacterial infections in hospitalized patients is community-acquired versus hospital-acquired?
5. What is the incidence of COVID-19-associated bacterial infection in un-hospitalized populations?
6. Do rates of COVID-19-associated bacterial infection vary by demographic categories, comorbidities, or geographic location?
7. What chronic damage does COVID-19 cause and in what organs? What are the implications of this damage for future bacterial infections and other health problems?
8. What are the mechanisms of bacterial infection associated with COVID-19?
9. What are the effects of antibiotic use in COVID-19 on overall resistance patterns?

Addressing these specific questions will help to move forward our understanding and management of COVID-19 both now and in the future.

## Data Availability Statement

The original contributions presented in the study are included in the article/Supplementary Material, further inquiries can be directed to the corresponding authors.

## Author Contributions

JF and SB contributed to the conception and design of the study. CZ conceptualized and performed the modeling. JF wrote the first draft of the manuscript. KT contributed to the clinical perspective and revision. All authors contributed to manuscript revision, and read and approved the submitted version.

## Conflict of Interest

The authors declare that the research was conducted in the absence of any commercial or financial relationships that could be construed as a potential conflict of interest.

## Footnotes

1 https://www.cdc.gov/coronavirus/2019-ncov/hcp/faq.html
