# Supplementary material for: Causes and Consequences of COVID-19-Associated Bacterial Infections
Source: Front Microbiol. 2021 Jul 20;12:682571. doi: 10.3389/fmicb.2021.682571 (PMC8329088; doi:10.3389/fmicb.2021.682571)
Supplement: Supplementary file 1 [file Image_1.pdf]

**SUPPLEMENT: simulation model of causal pathways**

In the main text results, we set the model tuning parameters *a*, *b*, and *c* to 1. Here we vary these parameters and examine model consistency with Zhou et al. (2020) data (Figure S1).

We simulate cohorts of 1000 hospitalized COVID-19 positive cases under a range of *a,b,c* scenarios, obtaining distributions for four possible outcomes: survivors with bacterial infection, survivors without bacterial infection, non-survivors with bacterial infection, and non-survivors without. We compare our modeled distributions to data from the Zhou et al study. Combinations of parameter values that were consistent with the data (within a 95% CI) are coded as green dots (Figure S1).

This model fitting shows that no parameter other than *b* can equal zero. This confirms our ruling out of the pipe structure (where *a* = 0) and allows us to rule out the fork structure (where *c* = 0) as very unlikely to be the true causal structure. Together, our models support both a collider or multi-path causal structure underpinning bacterial-associated mortality in COVID-19.

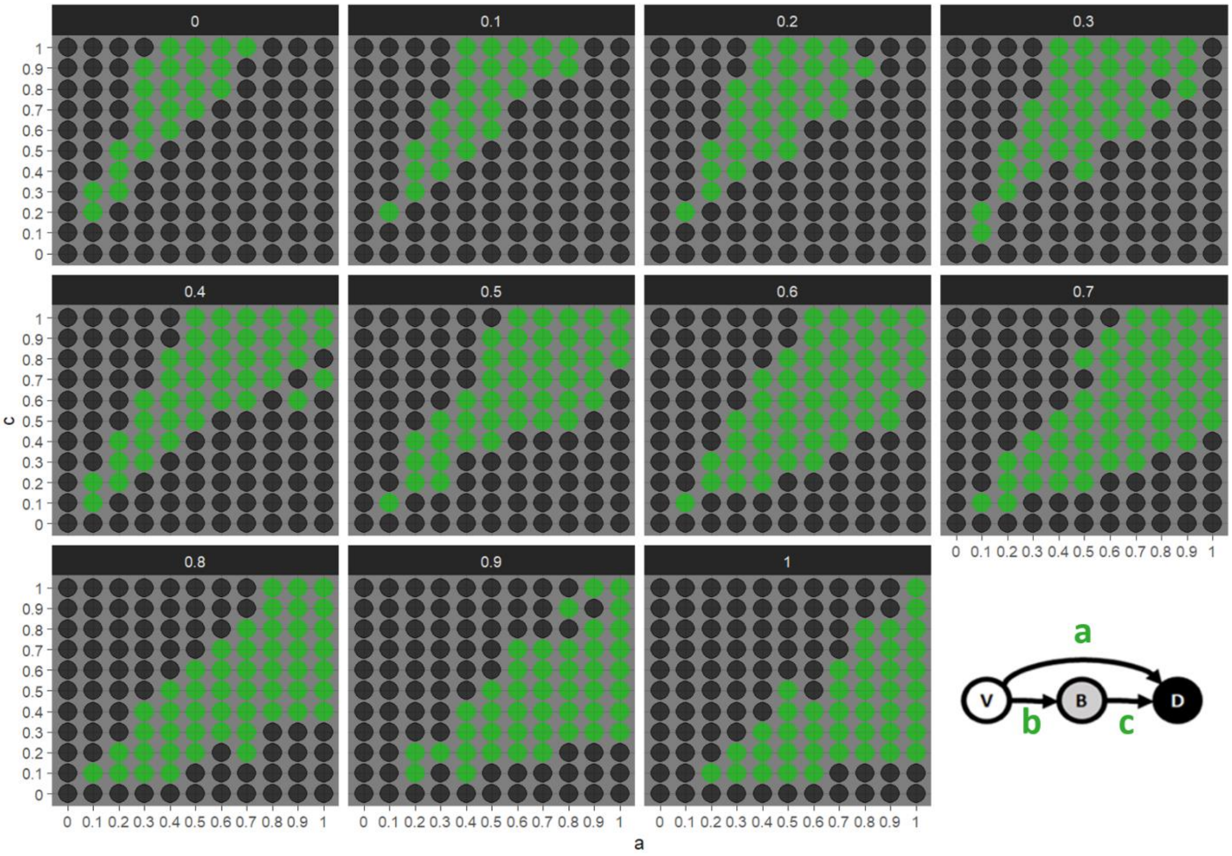


**Figure S1. Fitting the general model.** We fit a general model by introducing parameters *a*, *b*, and *c* to modulate the effect of (*V* → *D*), (*V* → *B*), and (*B* → *D*), respectively. For each combination of parameters *a*, *b*, and *c,* we run 100 simulations of cohorts of 1000 hospitalized patients each. For each cohort, obtaining distributions of the percentage of patients for each of four possible outcomes: survivors with bacterial infection, survivors without bacterial infection, non-survivors with bacterial infection, and non-survivors without. We then compare the observed percent distribution from the *Zhou et al* study to the simulated ones, and highlight where the observed percentage for each outcome falls within a 90% CI for the simulated distributions (green circles). Our model fitting shows that only b can equal zero and still be consistent with the observed infection and mortality data.
